# Activation of lignocellulosic biomass for higher sugar yields using aqueous ionic liquid at low severity process conditions

**DOI:** 10.1186/s13068-016-0561-7

**Published:** 2016-08-02

**Authors:** Ramakrishnan Parthasarathi, Jian Sun, Tanmoy Dutta, Ning Sun, Sivakumar Pattathil, N. V. S. N. Murthy Konda, Angelo Gabriel Peralta, Blake A. Simmons, Seema Singh

**Affiliations:** 1 Deconstruction Division, Joint BioEnergy Institute, Emeryville, CA 94608 USA; 2 Sandia National Laboratories, Biological and Engineering Sciences Center, Livermore, CA USA; 3 Complex Carbohydrate Research Center, University of Georgia, Athens, GA 30602 USA; 4 Oak Ridge National Laboratory, The BioEnergy Science Center, Oak Ridge, TN 37831 USA

**Keywords:** Aqueous ionic liquid, Pretreatment, Biofuels, Higher sugar yield, Low severity condition

## Abstract

**Background:**

Concerns around greenhouse gas emissions necessitate the development of sustainable processes for the production of chemicals, materials, and fuels from alternative renewable sources. The lignocellulosic plant cell walls are one of the most abundant sources of carbon for renewable bioenergy production. Certain ionic liquids (ILs) are very effective at disrupting the plant cell walls of lignocellulose, and generate a substrate that is effectively hydrolyzed into fermentable sugars. Conventional ILs are relatively expensive in terms of purchase price, and the most effective imidazolium-based ILs also require energy intensive processing conditions (>140 °C, 3 h) to release >90 % fermentable sugar yields after saccharification.

**Results:**

We have developed a highly effective pretreatment technology utilizing the relatively inexpensive IL comprised tetrabutylammonium [TBA]^+^ and hydroxide [OH]^−^ ions that generate high glucose yields (~95 %) after pretreatment at very mild processing conditions (50 °C). The efficiency of [TBA][OH] pretreatment of lignocellulose was further studied by analyzing chemical composition, powder X-ray diffraction for cellulose structure, NMR and SEC for lignin dissolution/depolymerization, and glycome profiling for cell wall modifications. Glycome profiling experiments and computational results indicate that removal of the noncellulosic polysaccharides occurs due to the ionic mobility of [TBA][OH] and is the key factor in determining pretreatment efficiency. Process modeling and energy demand analysis suggests that this [TBA][OH] pretreatment could potentially reduce the energy required in the pretreatment unit operation by more than 75 %.

**Conclusions:**

By leveraging the benefits of ILs that are effective at very mild processing conditions, such as [TBA][OH], lignocellulosic biomass can be pretreated at similar efficiency as top performing conventional ILs, such as 1-ethyl-3-methylimidazolium acetate [C_2_C_1_Im][OAc], but at much lower temperatures, and with less than half the IL normally required to be effective. [TBA][OH] IL is more reactive in terms of ionic mobility which extends removal of lignin and noncellulosic components of biomass at the lower temperature pretreatment. This approach to biomass pretreatment at lower temperatures could be transformative in the affordability and energy efficiency of lignocellulosic biorefineries.

**Electronic supplementary material:**

The online version of this article (doi:10.1186/s13068-016-0561-7) contains supplementary material, which is available to authorized users.

## Background

**Fig. 1:**
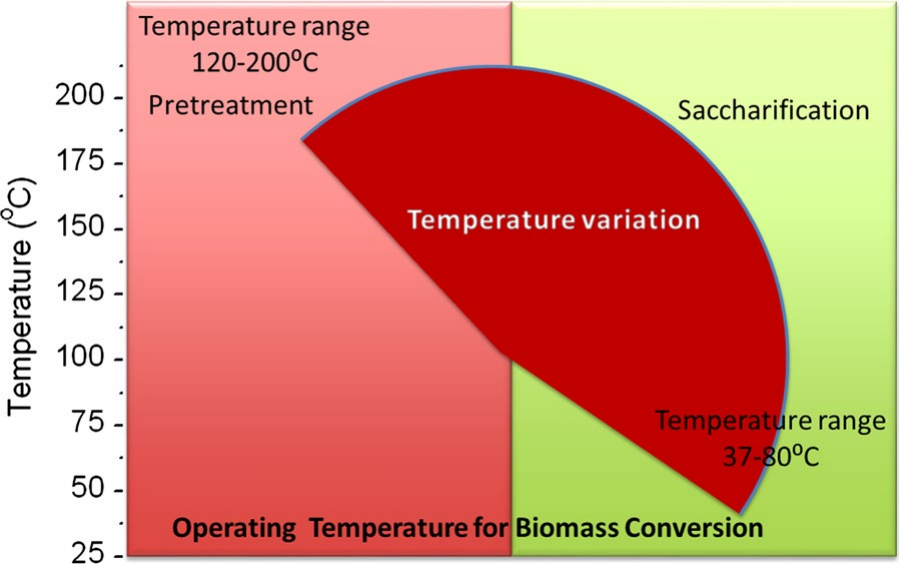
Temperature variations in a typical biomass pretreatment and saccharification processes

Lignocellulosic biomass is an abundant renewable resource primarily composed of cellulose, lignin, and hemicellulose that form a complex composite structure [1–4]. The recalcitrance of this complex composite poses a significant barrier to economical, chemical, or biological conversion technologies that can convert the fermentable sugars present in lignocellulose into advanced biofuels and renewable chemicals [5]. Several physical and/or chemical pretreatment processes have been implemented to reduce the recalcitrance of lignocellulosic materials and improve their utilization [6–8]. Conventional pretreatments, such as those that use concentrated or dilute acids and bases, are only effective in producing a substrate capable of generating high fermentable sugar yields using severe process conditions (~120–200 °C). After pretreatment, the recovered substrate is saccharified using enzyme mixtures at 40–80 °C. There is an intricate interplay between the type of pretreatment and fermentable sugar yields achieved (Fig. 1) [6, 9–15]. These higher temperature process technologies increase the energy required, and thereby increase production costs [7]. Generally, temperature of pretreatment process has been set around the range of the glass transition temperature of lignin, thereby impacting the physicochemical properties of lignin and cellulose [16], hemicellulose hydrolysis [17], and cellulose digestion [18].

Certain ionic liquids (ILs) are able to dissolve either lignocellulosic materials or one of its main constituents, such as cellulose, hemicellulose, or lignin [19]. The IL 1-ethyl-3-methylimidazolium acetate ([C_2_C_1_Im][OAc]) based pretreatment process typically requires temperatures above 140 °C for 3 h reaction time to be effective and typically use pure IL as the pretreatment solvent [10, 12, 20–22]. There have been recent efforts focused on the discovery and demonstration of ILs for the pretreatment/fractionation of lignocellulosic materials at less severe process conditions [23–25]. [OH] based ILs with [C_2_C_1_Im] were used for biochemical synthesis, such as sugars [26], biodiesel [27] and 5-hydroxymethylfurfural [26, 28]. Studies on quaternary ammonium cations, such as tetrabutylammonium fluoride trihydrate ([TBA]F) [29], tetraethylammonium chloride ([TEA]Cl) [30], and tetrabutylammonium hydroxides ([TBA][OH]) [31] with co-solvents, were reported to dissolve cellulose rapidly at low temperatures. This rapid dissolution of cellulose at low temperatures has been hypothesized to occur due to the strong proton accepting capacity of the anion, even in the presence of water that weakens the association of the hydrogen bonding network and destabilizing the cellulose microstructure.

Recent studies using tetrabutylammonium acetate ([TBA][OAc]) with dimethyl sulfoxide (DMSO) and crown ether (18-crown-6) demonstrated the feasibility of 8 wt% cellulose dissolution within 5 min at 40 °C [32]. Tetrabutylphosphonium hydroxides ([TBP][OH]) containing 30–50 wt% water can dissolve cellulose at 25 °C [33], and Ohno and co-workers recently reported the rapid (~5 min) dissolution of 15 wt% cellulose in aqueous solutions of [TBP][OH] that contained 40–50 % water by weight at room temperature [34]. Zhong et al. reported cellulose isolation from wheat straw using [TBA][OH] solutions containing 50 % water at 60 °C after de-waxed with toluene–ethanol (2:1, v/v) and pretreated in boiling water for 2 h [35]. Unfortunately, from a biorefinery perspective, all of these methods require multi-step treatments, use of co-solvents, water washes and have not been proven on a wide range of “real world” lignocellulosic biomass substrates, such as switchgrass, pine and eucalyptus. The possibility of IL pretreatment at lower operating temperatures may facilitate the development of more affordable and practical pretreatment processes with seamless biomass integrated conversion processes [3, 13, 36]. We report here that the relatively inexpensive [37] [TBA][OH] processing of lignocellulose can pretreat biomass to similar efficiency as top performing conventional ILs, such as [C_2_C_1_Im][OAc], but at much lower temperatures, and with less than half the IL normally required to be effective.

## Results and discussion

### Compositional analysis and lignin fractionation

The compositional analysis of switchgrass before and after pretreatment is summarized in Table 1. Solid recovery refers to the mass percentage of biomass (dry weight) recovered from the original biomass load. Three of the major plant cell wall components of switchgrass (i.e., glucan, xylan, and acid-insoluble lignin), were monitored before and after the pretreatment. Untreated dry switchgrass contained 31.9 % glucan, 20.2 % xylan and 20.7 % acid-insoluble lignin. The pretreatment experiments were conducted at different conditions (i.e., 25 °C for 0.5, 1 and 3 h; 50 °C for 0.5, 1 and 3 h). The solid recovery was obviously decreased with increasing temperature and time. Using the conditions of 50 °C for 3 h, approximately 48 wt% of the biomass was recovered, of which 62 % was glucan, 12.7 % was xylan and 10.5 % was lignin. Based on the compositional change, the mass loss is caused by significant removal of lignin, xylan, and/or other soluble extractives. While the loss of glucan was approximately 6.5 wt%, the removal of xylan was significantly higher (~69.8 wt%). Also, the lignin removal during pretreatment process (~75.7 wt%) was comparable with our previous results for switchgrass with 49–87 % lignin removal after pretreatment with different types ILs at high temperature (140 °C) [21].

**Table 1:** Compositional analysis of pretreated switchgrass and the removal of the major components^a^

Temp./time (°C/h)	Solid recovery	Composition of pretreated biomass			Removal of the major components^c^		
		Glucan (%)	Xylan (%)	Lignin (%)^b^	Glucan (%)	Xylan (%)	Lignin (%)^b^
/	/	31.9 ± 0.1	20.2 ± 0.1	20.7 ± 0.1	/	/	/
25/0.5	71.3 ± 0.5	43.8 ± 0.6	19.2 ± 0.1	16.1 ± 0.1	2.2	32.2	44.7
25/1	68.5 ± 0.5	45.5 ± 1.0	18.8 ± 0.4	16.3 ± 0.8	2.3	36.2	46.2
25/3	65.2 ± 0.4	47.7 ± 0.3	18.2 ± 0.1	13.0 ± 0.1	2.4	41.1	59.0
50/0.5	57.2 ± 0.5	54.4 ± 0.2	16.6 ± 0.2	13.8 ± 0.1	2.5	53.0	62.0
50/1	51.0 ± 0.3	59.5 ± 0.6	15.9 ± 0.3	12.4 ± 0.2	4.3	59.8	69.6
50/3	48.1 ± 0.4	62.0 ± 0.6	12.7 ± 0.1	10.5 ± 0.3	6.5	69.8	75.7

^a^The calculation is based on biomass dry weight

^b^ Acid-insoluble lignin

^c^ Removal of the major components is calculated based on the compositions of raw switchgrass

### Cellulose crystallinity

X-ray diffraction (XRD) studies were conducted to determine the changes in the crystalline *vs.* noncrystalline components (i.e., amorphous cellulose, hemicellulose and lignin) found in the switchgrass sample, and to monitor the structural changes in these polymers that occur during [TBA][OH] pretreatment. Commercial Avicel was used as cellulose standard to validate the results. Further, components isolated from the pretreatment condition (50 °C for 3 h) were utilized for cellulose crystallinity and lignin characterization studies.

Additional file 1: Fig. S1 shows the X-ray diffractograms of the untreated and pretreated switchgrass after processing at 50 °C for 3 h. The diffractogram obtained from the untreated switchgrass has two major diffraction peaks at 22.5° and 15.7° 2*θ*, characteristic of the cellulose I polymorph that corresponds to [002] and combined [101] + [10 $\bar{1}$] lattice planes, respectively. The third small peak at 34.5° ([040] lattice plane) corresponds to 1/4 of the length of one cellobiose unit and arises from ordering along the fiber direction [38–40]. Obtained crystallinity index from the XRD patterns of the pretreated switch grass indicates that the low temperature pretreatment based on 40 wt% [TBA][OH] has minimal impacts on cellulose. Although the diffractogram obtained from switchgrass pretreated with [TBA][OH] still retains the cellulose I polymorph, a small shift is observed in all three peaks. The diffractogram obtained from switchgrass pretreated with [TBA][OH] still retains the cellulose I polymorph with a realignment of [002] peak with Avicel indicating removal of amorphous cell wall components such as lignin and hemicellulose [21, 41]. This also reflected in the increase in the crystallinity index (CrI) of switchgrass from 67 to 76 % after pretreatment.

### Sugar yields

The sugar yields are calculated based on the glucan or xylan present in the original biomass (converting pretreated biomass to original using the solid recovery data in Table 1). As shown in Table 2, pretreatment using [TBA][OH] at 50 °C followed by saccharification generated high glucose yields of 93.1 % after 3 h, and pretreatment at 25 °C generated glucose yields of 72.2 % (0.5 h) to 76.9 % (3 h). Not surprisingly, longer pretreatment times resulted in better glucose yields for both temperatures. Regardless of operating time, a higher xylose yield after saccharification was obtained at the lower temperatures studied, and is attributed to more hemicellulose being present in the recovered solids after pretreatment. The enhancement of sugar yields at the lower temperature aqueous [TBA][OH] IL pretreatment is primarily due to the removal of hemicellulose and lignin. The results indicate that an aqueous solution of 40 wt% [TBA][OH] solution is efficient for the pretreatment of switchgrass at mild conditions. In addition, the recyclability of [TBA][OH] in biomass dissolution has been demonstrated recently [35]. Using the conditions (e.g., 50 °C, vacuum degree 0.1 MPa), it is expected that after lignin filtration, [TBA][OH] could be generated and reused for next run.

**Table 2:** Glucose and xylose yields after enzymatic saccharification of the pretreated switchgrass

Temp. (°C)^a^	Time/h	Glucose yield (%)^c^	SD	Xylose yield (%)^c^	SD
25^b^	–	15	2.0	10.0	2.0
25	0.5	72.2	0.1	31.2	0.1
25	1	76.2	0.2	36.4	0.1
25	3	76.9	0.3	34.3	0.1
50	0.5	85.6	0.3	26.4	0.1
50	1	86.7	0.6	28.1	0.1
50	3	93.1	2.54	20.6	1.34

^a^ Pretreatment temperature

^b^ Untreated original biomass

^c^ Calculation is based on the glucan or xylan present in the original biomass

### Lignin characterization

To examine the effect of [TBA][OH] pretreatment process on lignin, we carried out detailed lignin characterization studies using size exclusion chromatography (SEC) and 2D ^13^C-^1^H HSQC NMR techniques. The lignin solubilized in the [TBA][OH] after pretreatment (L_1_) was isolated by adjusting the pH to 2–3. The isolated lignin was compared with enzymatic mild acid lignin (EMAL) extracted from switchgrass, as it is commonly believed to be a close representation of the ‘native’ switchgrass lignin. The EMAL lignin from switchgrass was isolated based on the procedure reported by Wu and Argyropoulos [42]. The elution profiles acquired by monitoring UV absorbance (*λ* = 280 nm) from SEC measurements of EMAL and the lignin isolated from the liquid stream (L_1_) are depicted in Additional file 1: Fig. S2. Although the main elution peaks (*M*_w_ = 1.0–10.0 kDa) for both EMAL and L_1_ are comparable, a through comparison in the higher molecular weight region (*M*_w_ > 10.0 kDa) region shows that L_1_ has slightly lower molecular weight than EMAL, indicating small reduction of size. Apart from that L_1_ shows greater low molecular weight tails (*M*_w_ > 1.0 kDa) along with one new intense low molecular weight peak (*M*_w_ = 322 Da), both these observations indicate more abundant lower molecular weight lignin fractions as compared to EMAL.

**Fig. 2:**
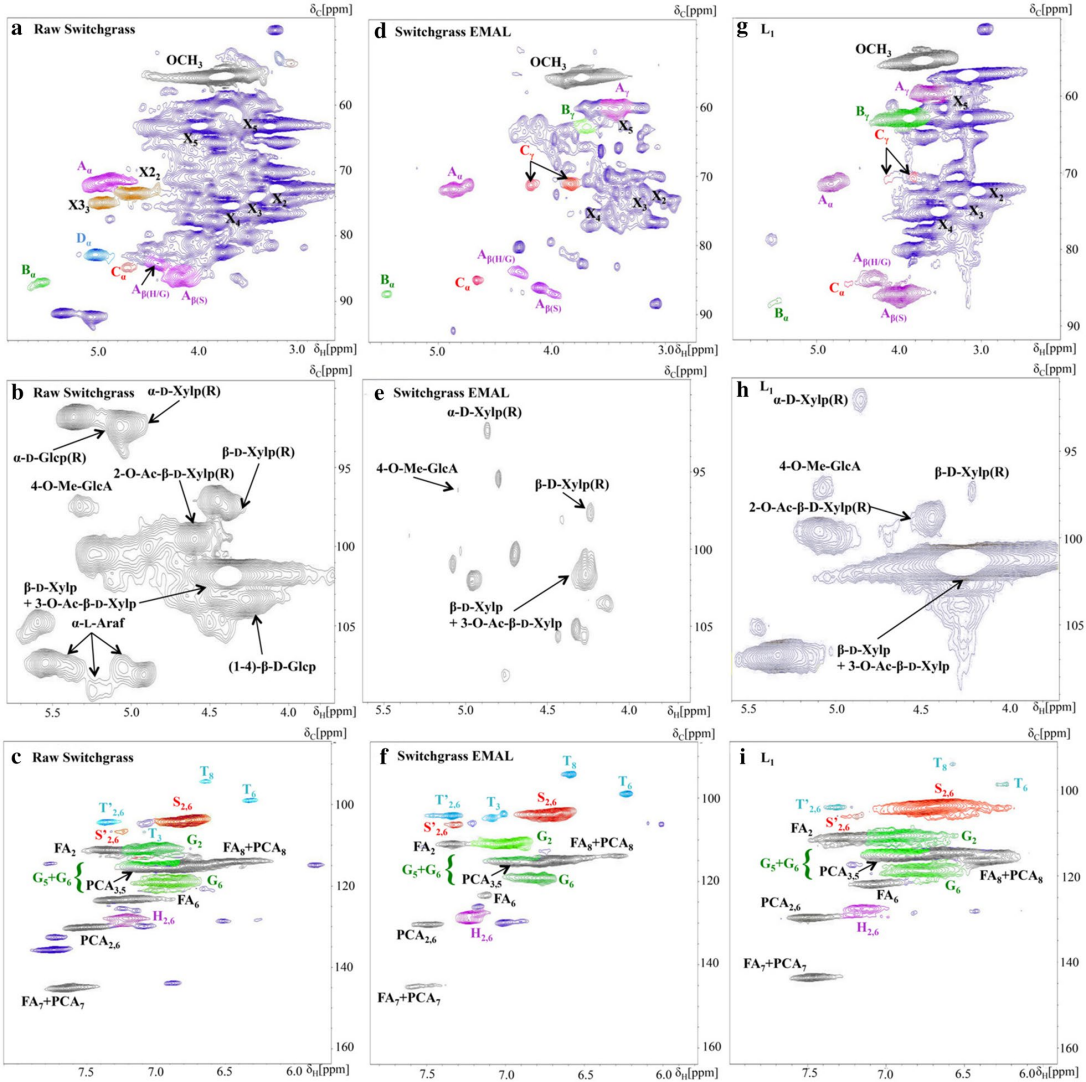
2D HSQC NMR spectra of aliphatic regions of untreated switchgrass (**a**), EMAL (**d**) and L_1_ (**g**); anomeric regions of untreated switchgrass (**b**), EMAL (**e**) and L_1_ (**h**); and aromatic regions of untreated switchgrass (**c**), EMAL (**f**) and L_1_ (**i**)

To understand the structural changes that occur in lignin during the pretreatment process, the isolated lignin was compared with EMAL and raw switchgrass using 2D ^13^C-^1^H heteronuclear single quantum coherence (HSQC) nuclear magnetic resonance (NMR) (Fig. 2). The cross peaks were assigned according to standards reported in the literature [43–49]. The structures in these HSQC spectra correspond to the color-coded structures depicted in Additional file 1: Fig. S3. Lignin side chains and interunit correlations are shown in aliphatic (top row, Fig. 2a) and aromatic regions (bottom row, Fig. 2c) and the polysaccharides are shown in the anomeric region (middle row, Fig. 2b). The relative changes in the lignin chemical structures were determined based on the volume integrations of HSQC spectral contour correlations. The HSQC spectrum of the cell wall of the untreated switchgrass shows that the β-aryl ether interunit linkages (A_α_, A_β(H/G)_, A_β(S)_, substructure A) are the predominant linkages in the lignin with small contributions of phenylcoumaran (β-5, substructure B), resinol (β–β, substructure C), and dibenzodioxocin (substructure D) linkages. The aliphatic region also exhibits two distinct peaks of 2-*O*-Ac-β-d-Xyl*p*(R) (X2_2_) and 3-*O*-Ac-β-d-Xyl*p*(R) (X3_3_) that represent major acetylated components of hemicelluloses. The aromatic region of the cell wall of the raw switchgrass indicates that the lignin is a S/G type lignin with a minor amount of H-type lignin containing *p*-coumarates (*p*CA) and ferulates (FA), which is consistent with previous literature reports [43, 46, 47, 49]. The HSQC spectrum of the untreated switchgrass also shows the presence of tricin moieties (substructure T).

The HSQC spectrum of L_1_ is shown in Fig. 2g–i. The weaker signal intensity of the A_α_ interunit linkages suggest chemical changes in the β-aryl ethers during the [TBA][OH] pretreatment. The absence of dibenzodioxocin signal (*δ*C/*δ*H: 83.3/4.81 ppm) indicates that the lignin isolated from the liquid stream is more linear as compared to the branched lignin in the untreated switchgrass due to removal of the points of branching [50]. Additionally, the absence of X2_2_ and X3_3_ correlations suggest deacetylation of hemicellulose occurred more readily at C_2_/H_2_ position. The anomeric regions of the untreated biomass and L_1_ demonstrates a noticeable decrease of α-d-Glcp(R)/α-d-Xlyp(R), may be due to glycosidic bond cleavage and reduction in the degree of polymerization (DP) of hemicellulose during [TBA][OH] pretreatment. When compared with the switchgrass EMAL (Fig. 2d–f), L_1_ has similar interunit traits in both aliphatic and aromatic regions of the HSQC spectra. The relative abundance of different interunit linkages in EMAL and L_1_ is shown in Additional file 1: Fig. S4. In L_1_, the β-aryl ether interunit linkages decrease from 59 to 43 % as compared to the EMAL, with a relatively smaller decrease in both phenylcoumaran and resinol substructures. This result is in agreement with the SEC results, confirming reduction of the lignin size due to depolymerization during [TBA][OH] pretreatment process. The absence of detectable dibenzodioxocin substructure suggests relatively linear lignin structure of both types of lignins. Tricin substructures were also detected in both EMAL and L_1_ [51]. The SEC and 2D NMR studies suggested that L_1_ has similar structure traits as switchgrass EMAL with a relatively smaller size. These results indicate that [TBA][OH] can very efficiently solubilize and partially depolymerize the lignin present in switchgrass.

### Glycome profiling

**Fig. 3:**
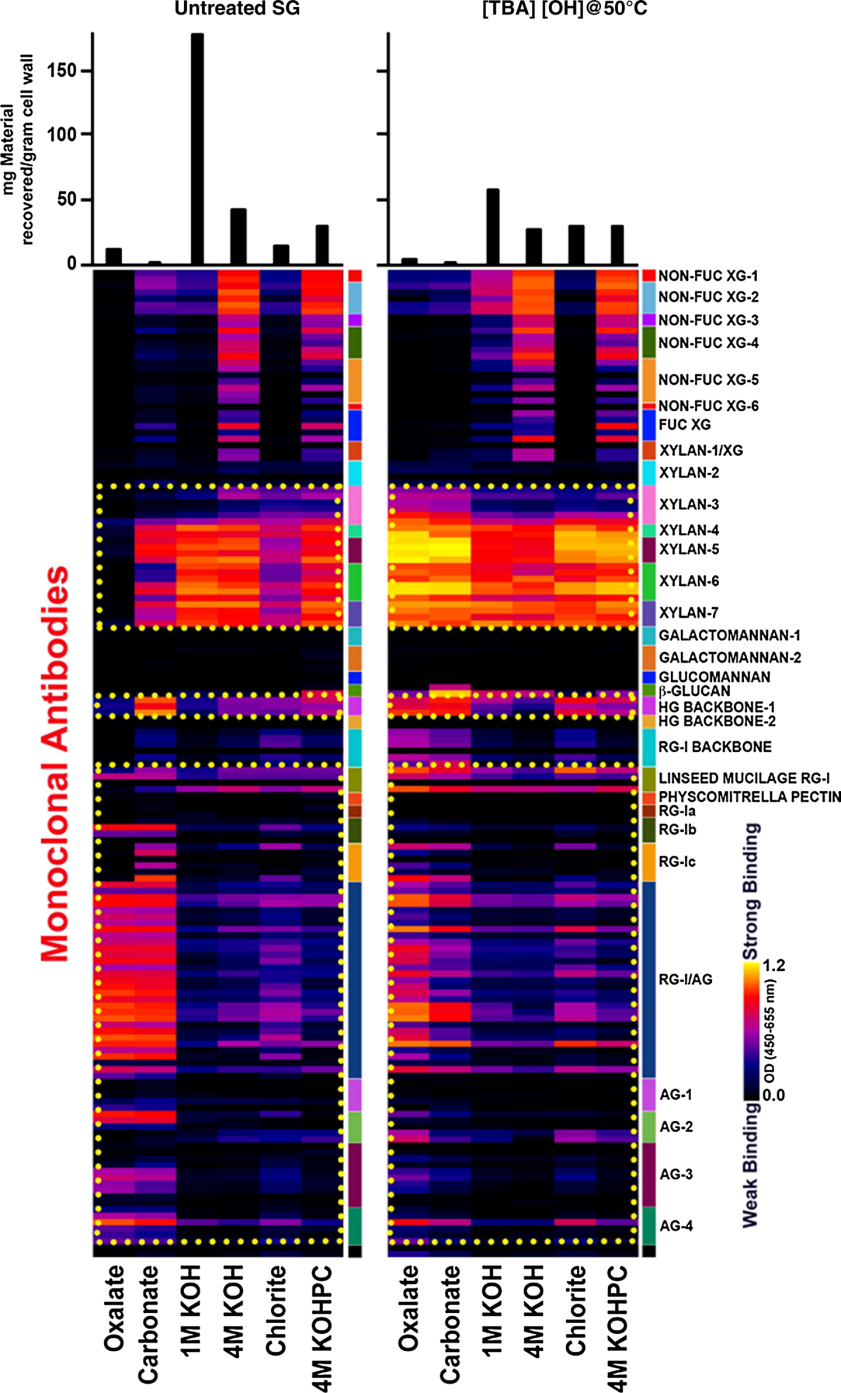
Glycome profiling of untreated switchgrass, and switchgrass pretreated with [TBA][OH] at 50 °C for 3 h: Sequential cell wall extracts (*bottom*) were subjected to ELISA screens with monoclonal antibodies for most major noncellulosic plant glycan classes (*right*). The ELISA binding response values are represented as a *color-coded* “heatmap” (*center*) and the recovered masses of carbohydrate material resulting from each extraction step is represented with *bar graphs* (*top*)

Glycome profiling of untreated and [TBA][OH] pretreated switchgrass biomass was conducted to facilitate a comparative study of the overall changes in composition and extractability patterns of the major noncellulosic cell wall glycans. Concomitantly, the results from glycome profiling analyses were employed to understand how [TBA][OH] pretreatment cause reduced cell wall recalcitrance in switchgrass. Glycome profiles of untreated and [TBA][OH] pretreated switchgrass at 50 °C, as depicted in Fig. 3, revealed significant differences that are highlighted in the yellow dotted blocks. These significant differences in the glycome profiles of pretreated biomass indicate that pretreatment regime caused an overall change in cell wall structure, architecture and composition. In general, [TBA][OH] pretreated switchgrass exhibited lesser amounts of materials recovered (see bar graphs on top panel) in all base extracts (1 M KOH, 4 M KOH and 4 M KOHPC) hinting at the removal and fragmentation of potentially noncellulosic cell wall components (that may also include hemicelluloses) during the [TBA][OH] pretreatment process. Interestingly, prominent variations were observed in the abundances of noncellulosic glycan epitopes in oxalate and carbonate extracts from pretreated biomass samples in comparison to the untreated biomass. For instance, a considerably higher abundance of xylan epitopes (as indicated by the higher binding of xylan-3 through xylan-7 groups of mAbs that recognize both unsubstituted and substituted xylans) was observed in oxalate and carbonate extracts from [TBA][OH] pretreated switchgrass at 50 °C that indicates enhanced xylan extractability. As previous studies have noted, such enhanced extractability of xylan epitopes is indicative of structural changes in the cell walls that results in reduced recalcitrance [52].

Substantial differences in patterns of extractabilities of epitopes of pectic components were also observed between the untreated and [TBA][OH] pretreated switchgrass. For example, oxalate extracts from [TBA][OH] pretreated samples showed significantly higher abundance of pectic backbone epitopes (as indicated by the higher binding of homogalacturonan backbone-1 and rhamnogalacturonan-I backbone groups of mAbs). Pectic arabinogalactan and arabinogalactan epitopes (as indicated by the binding of RG-I/AG and AG groups of mAbs) were highly abundant in oxalate and carbonate extracts from untreated switchgrass, however, this abundance decreased in [TBA][OH] pretreated switchgrass samples (potentially due to enhanced glycan fragmentation). The chlorite extraction step employed in sequential extraction breaks up and removes lignin to release any lignin-associated polysaccharides into the extract. Chlorite extracts from pretreated switchgrass under [TBA][OH] at 50 °C conditions contained higher abundance of xylan epitopes as compared to untreated samples indicating ILs perturbing effect on potential lignin-xylan associations. Overall, glycome profiling studies revealed the major structural modifications induced by [TBA][OH] at the mild pretreatment condition on switchgrass biomass resulting in efficient hydrolysis of cellulose to its constituent sugars.

### Computational modeling

**Fig. 4:**
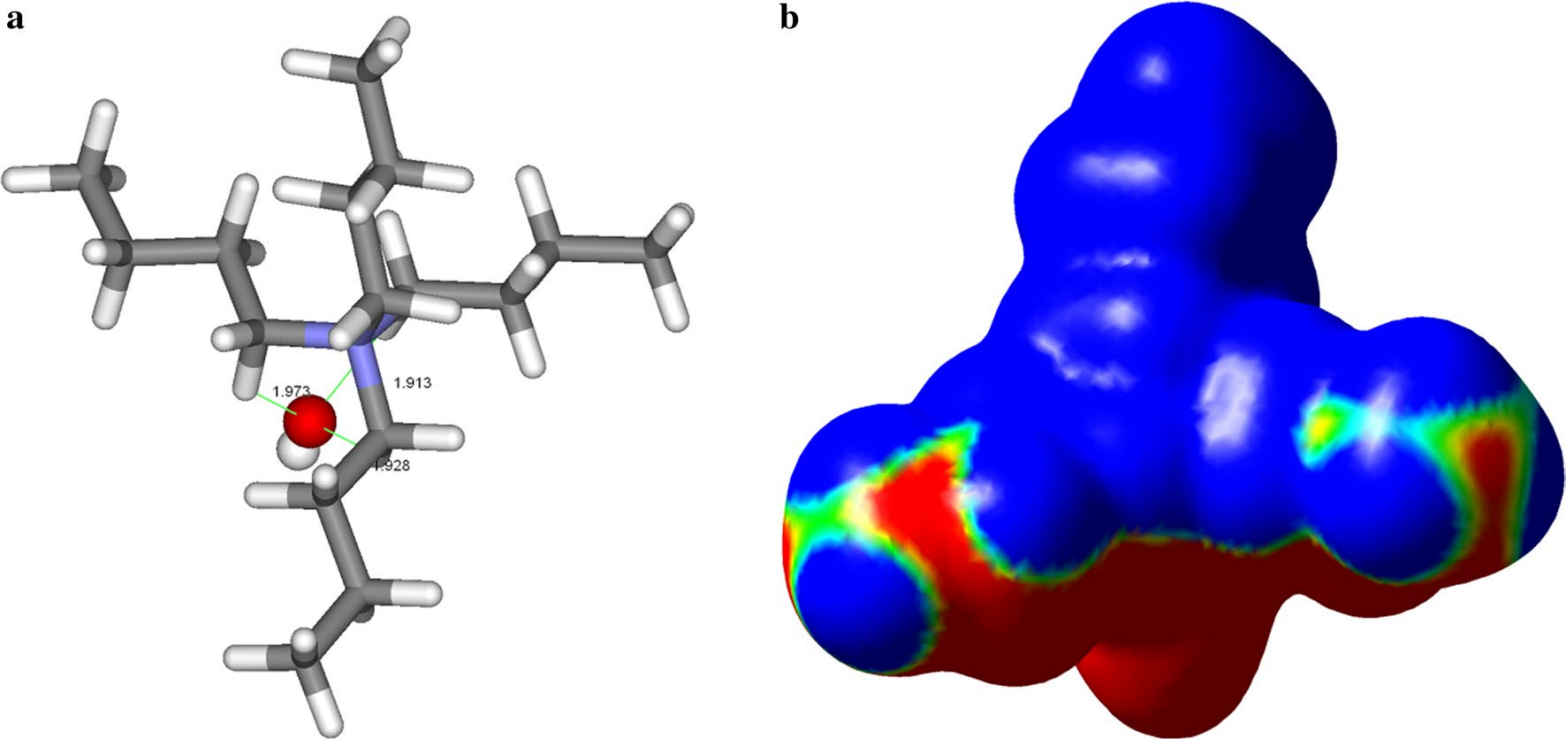
**a** Optimized geometry indicating anion cation association **b** molecular electrostatic potential map of [TBA][OH] at the ±0.04 au isosurface. The *color scale* indicates the charges on the atoms: *red* = most negative, *green* = neutral, *blue* = most positive charge

To understand the molecular level forces on the biomass dissolution at low temperature using [TBA][OH], the first requirement is to determine the chemical nature of [TBA][OH] that solubilizes lignocellulose. The optimized molecular geometry of [TBA][OH] obtained using hybrid density functional theory (DFT) calculations is shown in Fig. 4. The most stable conformation arises from interactions of an oxygen atom of the [OH]^−^ with the ionic region around nitrogen atom in [TBA]^+^. Due to the short O_OH_ … H-C_TBA_ bond distances (1.91–1.97 Ǻ) between cation and the anion of [TBA][OH] allowing strong intermolecular interaction energy (IE) (116.8 kcal/mol) of those ion pairs. It is clearly seen from the depicted molecular electrostatic potential (MESP) map of [TBA][OH] that the distinct separation of positive (blue) and negative regions (red) play a dominant role by influencing strong ionic interactions, electrostatics, and hydrophobic interactions with biomass components [53].

**Fig. 5:**
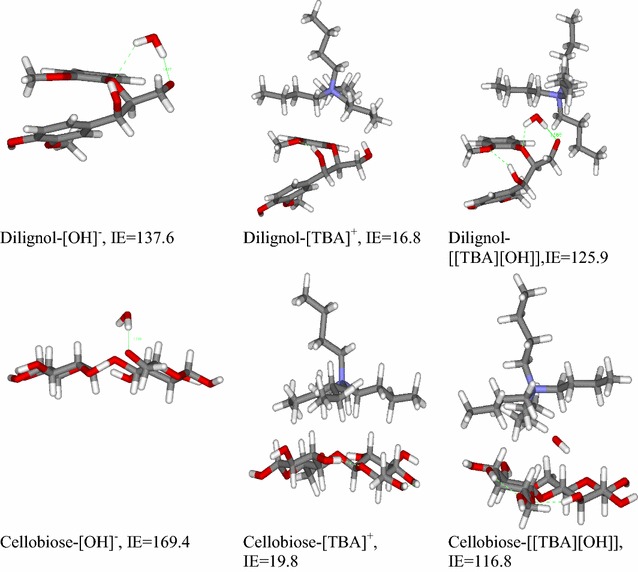
Optimized geometries of dilignol and cellobiose with [OH]^−^, [TBA]^+^ and [TBA][OH]. Interaction energy (IE) is reported in kcal/mol

We evaluated the influence of the anion and cation interactions on biomass dissolution by performing quantum chemical calculations of [OH]^−^, [TBA]^+^ and [TBA][OH] interacting with a model dilignol and cellobiose compounds (Fig. 5). From the calculated IEs, it was found that [OH]^−^ interacts with dilignol and cellobiose more strongly than [TBA]^+^ cation, and the IE of [OH]^−^ with cellobiose is slightly higher than that of its interactions with dilignol. In the case of [TBA]^+^ interactions, our calculations show a slightly higher IE for cellobiose than for dilignol, which are most probably due to hydrophobic interactions. Interestingly, IE strength of ion pair complexes with biomass components are more favored toward dilignol than cellobiose. This trend agrees with the experimental observation on lignin removal and provides insights on [TBA][OH] interactions with biomass components. Previous experimental studies [35] on [TBA][OH] that have been carried out on cellulose show strong contact with cellulose in the absence of any other competing biomass compounds (i.e., lignin). Considering the components in whole biomass, interactions with [TBA][OH] reveal that this IL has more affinity with lignin, and thus enables higher amount of lignin removal. Delignification of [TBA][OH] enhances the cellulose accessibility for enzymatic digestion. Another important point to note is that [TBA][OH] interactions could also account for biomass permeability as a result of the large amount of xylan removal by the [OH]^−^, since these types of bases are known to involve reactions under mild conditions [7]. It has been reported that alkaline ions could instigate the progression of following steps involving (i) cellulose swelling; (ii) internal surface area enhancement; (iii) changes in cellulose crystallinity; (iv) hemicellulose removal; (v) reducing the lignin and carbohydrate association; and (vi) disrupting the lignin structure by breaking its glycosidic ether bond. Hence, lignin cannot further act as a protective shield to the cellulose after lignin dissolution, consequently making cellulose more susceptible for degradation.

We then sought to determine what properties and interactions are dominant at these lower temperatures. Ionic conductivity, ion mobility, and viscosity of ILs are the key factors and vary based on ion type, size, charge, and temperature [54]. The higher pH (~14) of [TBA][OH] significantly influences the lignin removal [55]. The low temperature mechanism of biomass dissolution is particularly dependent on the ion exchange and contribution from ionic interactions between the biomass and ILs. Optimized geometries of lignin with [C_2_C_1_Im][OAc] and lignin with [TBA][OH] (Additional file 1: Fig. S5) are analyzed more carefully. It is interesting to point out that the proton/hydrogen exchange from the dilignol to [OH] anion has seen for the [TBA][OH]-lignin complexes. On the other hand, no significant elongation was observed for the O–H group of the lignin interacting with [OAc]^−^ of [C_2_C_1_Im][OAc]-lignin complexes. This ion mobility of [OH]^−^ enhances the ionic conductivity, and the [C_2_C_1_Im]^+^ is involved in stabilizing the complexes. Therefore, [TBA][OH] is more reactive than the other ILs used in pretreatment at higher temperature in terms of ionic interactions that extends their solvation capability at the lower temperature. Higher temperature conditions could manipulate these solvation properties for other ILs with different anions and cations. More importantly, ion pair type, size, charge, and co-solvents can be modified to design ILs with effective ionic conductivity and viscosity [56] for the efficient biomass dissolution under mild conditions.

### Process modeling and energy demand analysis on low temperature IL pretreatment

**Fig. 6:**
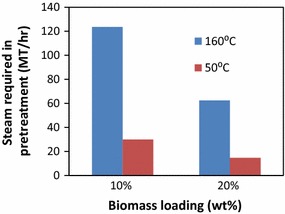
Impact of the temperature on energy requirement in the pretreatment process at industrial scale (to process 2000 MT/day dry biomass)

One important consideration for the developed pretreatment for [TBA][OH] is lower temperature process. To understand the impact of [TBA][OH] based low temperature pretreatment demonstrated in this study on the energy requirement, a process model was developed in SuperPro Designer (v8.5). The process consists of a pretreatment reactor and the performance of [TBA][OH] pretreatment process, in terms of energy intensity, is benchmarked against a more conventional [C_2_C_1_Im][OAc] process. The [TBA][OH] pretreatment was carried out at 50 °C while the [C_2_C_1_Im][OAc] pretreatment was conducted at 160 °C. Physical properties of the ILs (such as heat capacity) were collected from the literature [57]. Overall, four scenarios were constructed using these two ILs and two different biomass loadings (i.e., 10 and 20 %). Energy demand calculations revealed more than 75 % reduction in steam requirement during the low temperature pretreatment process to liberate carbohydrates with reduced energy input (Fig. 6) [36]. As shown in the Fig. 6, the impact of pretreatment temperature on energy requirement is lower for [TBA][OH] in both biomass loading conditions compared to [C_2_C_1_Im][OAc]. This effort could potentially address the challenging issues of developing robust and energy efficient technologies for increasing the sugar yields at a lower cost from renewable, nonfood lignocellulosic biomass.

## Conclusions

We have demonstrated the low temperature pretreatment of lignocellulosic biomass using the IL [TBA][OH] in the presence of water. The concept of inducing labile biomass deconstruction with reduced energy input and minimal IL loading has been successfully demonstrated. The effective pretreatment of 10 wt% switchgrass using aqueous mixtures of [TBA][OH] at 50 °C generated >90 % glucose yields and outperformed current best IL pretreatment based on imidazolium ILs at similar severities. Process modeling and energy demand analysis has shown significant potential for reduction in the pretreatment energy requirement. For instance, this results in more than a 75 % reduction in the steam requirement for [TBA][OH] compared to ILs that are used at temperatures above 140 °C. Compositional analysis of the [TBA][OH] pretreated switchgrass show that the enhancement of sugar yields at lower temperatures is due to the removal of hemicellulose and lignin. Lignin characterization using SEC and NMR on the extracted lignin after pretreatment indicates that [TBA][OH] efficiently solubilizes and partially depolymerizes the lignin during pretreatment. DFT computations on chemical reactivity of [TBA][OH] and its interaction with cellobiose and lignin reveal that [TBA][OH] IL is more reactive than the imidazolium IL used in pretreatment at higher temperature in terms of ionic mobility which enables lignin removal at the lower temperature. Glycome profiling experiments provide evidence for significant removal of noncellulosic components of biomass under mild [TBA][OH] pretreatment that is distinct from other ILs that require a higher temperature for better performance. By leveraging the benefits of ILs that are effective at very mild processing conditions, such as [TBA][OH], this study opens up an avenue for novel process designs that could significantly enhance the energy efficiency and affordability of the biorefinery by overcoming the temperature mismatch of pretreatment and saccharification unit operations.

## Methods

### Materials

Switchgrass (*Panicum virgatum*) was kindly provided from the laboratory of Prof. Daniel Putnam at the University of California, Davis. The switchgrass studied was a combination of lowland and upland varieties, grown in Davis, California and harvested in 2011. The samples were ground using a Thomas-Wiley® Mill fitted with a 20-mesh screen (Model 3383-L10 Arthur H. Thomas Co., Philadelphia, PA, USA) and used without further sieving. The samples were stored at 4 °C in a sealed plastic bag for use in all experiments. Commercial enzyme cocktails Cellic^®^ CTec 2 and HTec 2 were generously provided by Novozymes (Davis, CA). The ILs [TBA][OH] (>95 % purity) were purchased from Sigma-Aldrich.

### Biomass pretreatment

A 10 % (w/w) biomass solution was prepared by combining 1 g of switchgrass with 9 g of IL in a 25 mL tube reactor. The reactor was heated in an oil bath to the desired temperature and stirred at 150 rpm with a magnetic stir bar for 3 h. All pretreatment reactions were conducted in duplicate. Following pretreatment, 30 mL of deionized (DI) water was slowly added to the biomass/IL slurry with continued stirring. The mixture was transferred to 25 mL Falcon tubes and centrifuged at high speed (14,000 rpm) to separate solids. The pretreated biomass was further washed with 4 × 30 mL of DI water to remove any residual IL. The solids were lyophilized and stored at 4 °C for analysis.

### Compositional analysis

Compositional analysis of switchgrass before and after pretreatment was performed using NREL acidolysis protocols (LAP) LAP-002 and LAP-005. Briefly, 200 mg of biomass and 2 mL 72 % H_2_SO_4_ were incubated at 30 °C while shaking at 300 rpm for 1 h. The solution was diluted to 4 % H_2_SO_4_ with 56 mL of DI water and autoclaved for 1 h at 121 °C. The reaction was quenched by placing samples into an ice bath before removing the biomass by filtration. The filtrate was neutralized with CaCO_3_ and monomeric sugars were determined from the filtrate by Agilent HPLC 1200 Series equipped with a Bio-Rad Aminex HPX-87P column and a refractive index detector (aqueous mobile phase, 0.6 mL/min, column temperature 85 °C). The injection volume was 10 µL with a run time of 25 min. Acid-insoluble lignin was quantified gravimetrically from the solid after heating overnight at 105 °C (the weight of acid-insoluble lignin + ash) and then 575 °C for at least 6 h (the weight of ash).

### Enzymatic saccharification

Enzymatic saccharification of pretreated and untreated biomass was carried out using commercially available enzymes, Cellic^®^ CTec2 and HTec2 from Novozymes, at 50 °C, pH 5.5, and rotation speed of 150 rpm in a rotary incubator (Enviro-Genie, Scientific Industries, Inc.). All reactions were conducted at 10 % biomass loading by placing 500 mg of biomass (dry weight) in a 25 mL centrifuge tube. The pH of the mixture was adjusted to 5.5 with 50 mM sodium citrate buffer (pH 4.8) supplemented with 0.02 % NaN3 to prevent microbial contamination. The total reaction volume (5 mL) included a total protein content of 10 mg protein/g biomass (before pretreatment). The ratio of CTec2:HTec2 mixtures were held constant at 9:1 for all reactions. Reactions were monitored by centrifuging 50 µL aliquots of supernatant (5 min, 14,000 rpm) at specific time intervals and measuring monomeric sugar concentrations by HPLC as described previously.

### X-ray diffraction (XRD)

The raw and pretreated biomass/Avicel were dried and characterized with powder X-ray diffraction (PXRD). The XRD analysis were performed on a PANalytical Empyrean X-ray diffractometer equipped with a PIXcel^3D^ detector and operated at 45 kV and 40 kA using Cu Kα radiation (*λ* = 1.5418 Å). The patterns are collected in the 2*θ* range from 5° to 60° with a step size of 0.039° and the exposure time of 300 s. A reflection-transmission spinner was used as a sample holder and the spinning rate was set at 8 rpm throughout the experiment. Crystallinity index (CrI) was determined by Segal’s method [58].

### 2D ^13^C-^1^H HSQC NMR spectroscopy

Switchgrass cell wall and solids recovered from the liquid stream [TBA][OH] IL pretreatment via adjusting the pH were ball-milled, solubilized in DMSO-*d6*, and then analyzed by two-dimensional (2D) ^13^C–^1^H heteronuclear single quantum coherence (HSQC) nuclear magnetic resonance (NMR) as previously described [46]. Briefly, ball-milled samples (~50 mg) were placed in NMR tubes with 600 μl DMSO-*d6*. The samples were sealed and sonicated until homogeneous in a Branson 2510 table-top cleaner Branson Ultrasonic Corporation, Danburt, CT). The temperature of the bath was closely monitored and maintained below 50 °C. HSQC spectra were acquired at 398 K using a Bruker Avance-600 MHz instrument equipped with a 5 mm inverse gradient ^1^H/^13^C cryoprobe using the q_hsqcetgp pulse program (ns = 64, ds = 16, number of increments = 256, d1 = 1.5 s). Chemical shifts were referenced to the central DMSO peak (*δ*C/*δ*H 39.5/2.5 ppm). Assignment of the HSQC spectra is described elsewhere. A semi-quantitative analysis of the volume integrals of the HSQC correlation peaks was performed using Bruker’s Topspin 3.1 processing software.

### Size exclusion chromatography (SEC)

The molecular weight distribution of lignin was investigated using a gel permeation chromatography (GPC). The lignin was acetylated with pyridine and acetic anhydride following a previously published procedure [59]. The acetylated lignin was dissolved in tetrahydrofuran (THF) with a concentration of 1 g/L. GPC analysis was performed using a Tosoh Ecosec HLC-8320 GPC equipped with a refractive index (RI) and diode array detector (DAD) detector. Separation was achieved with an Agilent PLgel 5 μm Mixed-D column at 35 °C using a mobile phase of THF at a flow rate of 1.0 mL/min. The GPC standards, which contained polystyrene ranging from 162 to 29,150 g/mol, were purchased from Agilent and used for calibration. Absorbance of materials eluting from the column was detected at 280 nm (UV). The enzymatic mild acidolysis lignin (EMAL) process was used to extract lignin from switchgrass and it was used as a control.

### Computational details

The geometry optimizations of [TBA] cations and hydroxide anions, cellobiose, lignin dimer model (dilignol with *β*-*O*-4 linkage between two arene rings) were performed using density functional theory (DFT) with the M06-2X hybrid exchange–correlation functional and the 6-311 ++G(d, p) basis set. Frequency calculations were carried out to verify that the computed structures corresponded to energy minima. The most stable isolated cation/anion and their IL complexes obtained from our calculations are herein described. Several complexes of anions and cations interacting with cellobiose and dilignol (guided by the electrostatic potentials) were constructed and optimized at M06-2X/6-31G (d, p) basis set. The most stable complexes of cation and anion with cellobiose and dilignol were used to calculate interaction energies (IEs) at M06-2X/6-311 ++G(d, p) level using the supermolecular approach,

1 $$ \text{IE} = -\left( {E_{{\text{Complex}}} - \sum\limits_{i = 1}^{n} {E_{i} } } \right) $$

where *E*_Complex_ refer to the energies of cation and anion pair (for IL), anion or cation with biomass components, anion and cation with biomass complexes, respectively, and *E*_i_ refer to the energies of monomers. The results were corrected for basis set superposition error (BSSE) following the procedure adopted by Boys and Bernardi [60]. All quantum chemical calculations were performed using the Gaussian 09 suite of programs [Frisch et al. (2009) Gaussian 09 (Gaussian, Inc, Wallingford, CT), Revision D.01.] [61].

### Glycome profiling

Glycome profiling of raw and [TBA][OH] IL pretreated biomass samples that involves preparation of cell walls [Alcohol Insoluble Residues (AIR)], sequential extractions of AIR were carried out as previously described [62, 63]. Plant cell wall glycan-directed monoclonal antibodies (mAbs) were from laboratory stocks (CCRC, JIM and MAC series) at the Complex Carbohydrate Research Center (available through CarboSource Services; http://www.carbosource.net) or were obtained from BioSupplies (Australia) (BG1, LAMP). Supporting information on mAbs [64] used in this study can be found in the Supplementary Information Table S1, including the link to WallMabDB (http://www.wallmabdb.net) that provides detailed information for each antibody.

## Additional file

10.1186/s13068-016-0561-7 Detailed list of cell wall glycan-directed monoclonal antibodies (mAbs) used for glycome profiling analyses. The groupings of antibodies are based on a hierarchical clustering of ELISA data generated from a screen of all mAbs against a comprehensive panel of plant polysaccharide preparations that clusters mAbs according to the predominant polysaccharides that they recognize. The majority of listings link to the Wall*Mab*DB plant cell wall monoclonal antibody database (http://www.wallmabdb.net) that provides detailed descriptions of each mAb, including immunogen, antibody isotype, epitope structure (to the extent known), supplier information, and related literature citations. **Figure S1.** X-ray diffraction patterns and CrI (%) values of untreated Avicel, switchgrass solids and pretreated switch grass by [TBA][OH] at 50 °C pretreatment conditions. **Figure S2.** Normalized SEC chromatograms of EMAL- switchgrass and L_1_. **Figure S3.** HSQC spectra correspond to the color-coded substructures. **Figure S4.** Relative abundances in lignin interunit linkages in switchgrass EMAL and L_1_. **Figure. S5.** Optimized geometries of dilignol with IL complexes. (DOCX 697 kb).

## Abbreviations

IL: ionic liquid
[TBA]: tetrabutylammonium
[OH]: hydroxide
[C_2_C_1_Im][OAc]: 1-ethyl-3-methylimidazolium acetate
[TBA]F: tetrabutylammonium fluoride trihydrate
[TEA]Cl: tetraethylammonium chloride
[TBA][OAc]: tetrabutylammonium acetate
DMSO: dimethyl sulfoxide
[TBP][OH]: tetrabutylphosphonium hydroxide
XRD: X-ray diffraction
EMAL: enzymatic mild acid lignin
HSQC: heteronuclear single quantum coherence
NMR: nuclear magnetic resonance
SEC: size exclusion chromatography
GPC: gel permeation chromatography
BSSE: basis set superposition error
