# Supplementary material for: Activation of lignocellulosic biomass for higher sugar yields using aqueous ionic liquid at low severity process conditions
Source: Biotechnol Biofuels. 2016 Aug 2;9:160. doi: 10.1186/s13068-016-0561-7 (PMC4969646; doi:10.1186/s13068-016-0561-7)
Supplement: Supplementary file 1 — 10.1186/s13068-016-0561-7 Detailed list of cell wall glycan-directed monoclonal antibodies (mAbs) used for glycome profiling analyses. The groupings of antibodies are based on a hierarchical clustering of ELISA data generated from a screen of all mAbs against a comprehensive panel of plant polysaccharide preparations that clusters mAbs according to the predominant polysaccharides that they recognize. The majority of listings link to the WallMabDB plant cell wall monoclonal antibody database (http://www.wallmabdb.net) that provides detailed descriptions of each mAb, including immunogen, antibody isotype, epitope structure (to the extent known), supplier information, and related literature citations. Figure S1. X-ray diffraction patterns and CrI (%) values of untreated Avicel, switchgrass solids and pretreated switch grass by [TBA][OH] at 50 °C pretreatment conditions. Figure S2. Normalized SEC chromatograms of EMAL- switchgrass and L1. Figure S3. HSQC spectra correspond to the color-coded substructures. Figure S4. Relative abundances in lignin interunit linkages in switchgrass EMAL and L1. Figure. S5. Optimized geometries of dilignol with IL complexes. (DOCX 697 kb). [file 13068_2016_561_MOESM1_ESM.docx]

**Supporting Figures**

**Figure S1.** X-ray diffraction patterns and CrI (%) values of untreated Avicel, switchgrass solids and pretreated switch grass by [TBA][OH] at 50⁰C pretreatment conditions.

**Figure S2.** Normalized SEC chromatograms of EMAL- switchgrass and L_1._

**Figure S3.** HSQC spectra correspond to the color coded substructures.

**Supporting Fig. S4.** Relative abundances in lignin interunit linkages in switchgrass EMAL and L_1_.

| 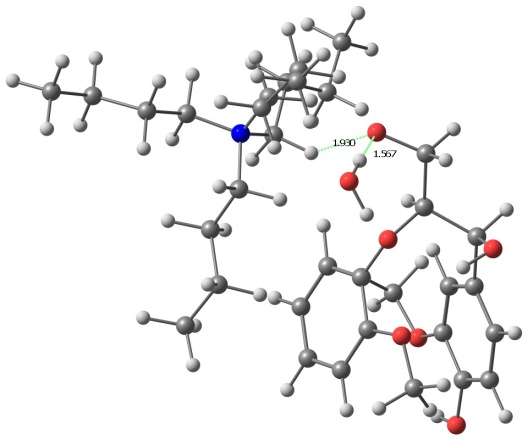 | 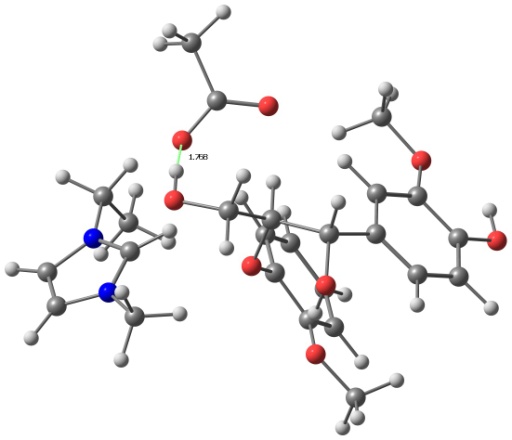 |
| --- | --- |
| [TBA][OH]— dilignol | [C_2_C_1_Im] [OAc]— dilignol |

**Figure S5.** Optimized geometries of dilignol with IL complexes.

**Table S1:** Detailed list of cell wall glycan-directed monoclonal antibodies (mAbs) used for glycome profiling analyses. The groupings of antibodies are based on a hierarchical clustering of ELISA data generated from a screen of all mAbs against a comprehensive panel of plant polysaccharide preparations[1, 2] that clusters mAbs according to the predominant polysaccharides that they recognize. The majority of listings link to the Wall*Mab*DB plant cell wall monoclonal antibody database (<http://www.wallmabdb.net>) that provides detailed descriptions of each mAb, including immunogen, antibody isotype, epitope structure (to the extent known), supplier information, and related literature citations.

**Glycan Group Recognized mAb Names**

| Non-Fucosylated Xyloglucan-1 | [CCRC-M95](http://glycomics.ccrc.uga.edu/wall2/jsp/abdetails.jsp?abnumber=162&abname=CCRC-M95) |
| --- | --- |
|  | [CCRC-M101](http://glycomics.ccrc.uga.edu/wall2/jsp/abdetails.jsp?abnumber=163&abname=CCRC-M101) |
|  |  |
|  |  |
| Non-Fucosylated Xyloglucan-2 | [CCRC-M104](http://glycomics.ccrc.uga.edu/wall2/jsp/abdetails.jsp?abnumber=164&abname=CCRC-M104) |
|  | [CCRC-M89](http://glycomics.ccrc.uga.edu/wall2/jsp/abdetails.jsp?abnumber=160&abname=CCRC-M89) |
|  | [CCRC-M93](http://glycomics.ccrc.uga.edu/wall2/jsp/abdetails.jsp?abnumber=161&abname=CCRC-M93) |
|  | [CCRC-M87](http://glycomics.ccrc.uga.edu/wall2/jsp/abdetails.jsp?abnumber=158&abname=CCRC-M87) |
|  | [CCRC-M88](http://glycomics.ccrc.uga.edu/wall2/jsp/abdetails.jsp?abnumber=159&abname=CCRC-M88) |
|  |  |
|  |  |
| Non-Fucosylated Xyloglucan-3 | [CCRC-M100](http://glycomics.ccrc.uga.edu/wall2/jsp/abdetails.jsp?abnumber=114&abname=CCRC-M100) |
|  | [CCRC-M103](http://glycomics.ccrc.uga.edu/wall2/jsp/abdetails.jsp?abnumber=113&abname=CCRC-M103) |
|  |  |
|  |  |
| Non-Fucosylated Xyloglucan-4 | [CCRC-M58](http://glycomics.ccrc.uga.edu/wall2/jsp/abdetails.jsp?abnumber=155&abname=CCRC-M58) |
|  | [CCRC-M86](http://glycomics.ccrc.uga.edu/wall2/jsp/abdetails.jsp?abnumber=157&abname=CCRC-M86) |
|  | [CCRC-M55](http://glycomics.ccrc.uga.edu/wall2/jsp/abdetails.jsp?abnumber=148&abname=CCRC-M55) |
|  | [CCRC-M52](http://glycomics.ccrc.uga.edu/wall2/jsp/abdetails.jsp?abnumber=145&abname=CCRC-M52) |
|  | [CCRC-M99](http://glycomics.ccrc.uga.edu/wall2/jsp/abdetails.jsp?abnumber=152&abname=CCRC-M99) |
|  |  |
|  |  |
| Non-Fucosylated Xyloglucan-5 | [CCRC-M54](http://glycomics.ccrc.uga.edu/wall2/jsp/abdetails.jsp?abnumber=147&abname=CCRC-M54) |
|  | [CCRC-M48](http://glycomics.ccrc.uga.edu/wall2/jsp/abdetails.jsp?abnumber=77&abname=CCRC-M48) |
|  | [CCRC-M49](http://glycomics.ccrc.uga.edu/wall2/jsp/abdetails.jsp?abnumber=76&abname=CCRC-M49) |
|  | [CCRC-M96](http://glycomics.ccrc.uga.edu/wall2/jsp/abdetails.jsp?abnumber=151&abname=CCRC-M96) |
|  | [CCRC-M50](http://glycomics.ccrc.uga.edu/wall2/jsp/abdetails.jsp?abnumber=143&abname=CCRC-M50) |
|  | [CCRC-M51](http://glycomics.ccrc.uga.edu/wall2/jsp/abdetails.jsp?abnumber=144&abname=CCRC-M51) |
|  | [CCRC-M53](http://glycomics.ccrc.uga.edu/wall2/jsp/abdetails.jsp?abnumber=146&abname=CCRC-M53) |
|  |  |
|  |  |
| Non-Fucosylated Xyloglucan-6 | [CCRC-M57](http://glycomics.ccrc.uga.edu/wall2/jsp/abdetails.jsp?abnumber=154&abname=CCRC-M57) |
|  |  |
|  |  |
| Fucosylated Xyloglucan | [CCRC-M102](http://glycomics.ccrc.uga.edu/wall2/jsp/abdetails.jsp?abnumber=142&abname=CCRC-M102) |
|  | [CCRC-M39](http://glycomics.ccrc.uga.edu/wall2/jsp/abdetails.jsp?abnumber=78&abname=CCRC-M39) |
|  | [CCRC-M106](http://glycomics.ccrc.uga.edu/wall2/jsp/abdetails.jsp?abnumber=112&abname=CCRC-M106) |
|  | [CCRC-M84](http://glycomics.ccrc.uga.edu/wall2/jsp/abdetails.jsp?abnumber=124&abname=CCRC-M84) |
|  | [CCRC-M1](http://glycomics.ccrc.uga.edu/wall2/jsp/abdetails.jsp?abnumber=1&abname=CCRC-M1) |
|  |  |
|  |  |
| Xylan-1/XG | [CCRC-M111](http://glycomics.ccrc.uga.edu/wall2/jsp/abdetails.jsp?abnumber=168&abname=CCRC-M111) |
|  | [CCRC-M108](http://glycomics.ccrc.uga.edu/wall2/jsp/abdetails.jsp?abnumber=149&abname=CCRC-M108) |
|  | [CCRC-M109](http://glycomics.ccrc.uga.edu/wall2/jsp/abdetails.jsp?abnumber=150&abname=CCRC-M109) |
|  |  |
|  |  |
| Xylan-2 | [CCRC-M119](http://glycomics.ccrc.uga.edu/wall2/jsp/abdetails.jsp?abnumber=106&abname=CCRC-M119) |
|  | [CCRC-M115](http://glycomics.ccrc.uga.edu/wall2/jsp/abdetails.jsp?abnumber=110&abname=CCRC-M115) |
|  | [CCRC-M110](http://glycomics.ccrc.uga.edu/wall2/jsp/abdetails.jsp?abnumber=167&abname=CCRC-M110) |
|  | [CCRC-M105](http://glycomics.ccrc.uga.edu/wall2/jsp/abdetails.jsp?abnumber=165&abname=CCRC-M105) |
|  |  |
|  |  |
| Xylan-3 | [CCRC-M117](http://glycomics.ccrc.uga.edu/wall2/jsp/abdetails.jsp?abnumber=108&abname=CCRC-M117) |
|  | [CCRC-M113](http://glycomics.ccrc.uga.edu/wall2/jsp/abdetails.jsp?abnumber=171&abname=CCRC-M113) |
|  | [CCRC-M120](http://glycomics.ccrc.uga.edu/wall2/jsp/abdetails.jsp?abnumber=105&abname=CCRC-M120) |
|  | [CCRC-M118](http://glycomics.ccrc.uga.edu/wall2/jsp/abdetails.jsp?abnumber=107&abname=CCRC-M118) |
|  | [CCRC-M116](http://glycomics.ccrc.uga.edu/wall2/jsp/abdetails.jsp?abnumber=109&abname=CCRC-M116) |
|  | [CCRC-M114](http://glycomics.ccrc.uga.edu/wall2/jsp/abdetails.jsp?abnumber=111&abname=CCRC-M114) |
|  |  |
| Xylan-4 | CCRC-M154 |
|  | CCRC-M150 |
|  |  |
|  |  |
| Xylan-5 | CCRC-M144 |
|  | CCRC-M146 |
|  | CCRC-M145 |
|  | CCRC-M155 |
|  |  |
|  |  |
| Xylan-6 | CCRC-M153 |
|  | CCRC-M151 |
|  | CCRC-M148 |
|  | CCRC-M140 |
|  | CCRC-M139 |
|  | CCRC-M138 |
|  |  |
|  |  |
| Xylan-7 | CCRC-M160 |
|  | [CCRC-M137](http://glycomics.ccrc.uga.edu/wall2/jsp/abdetails.jsp?abnumber=173&abname=CCRC-M137) |
|  | CCRC-M152 |
|  | CCRC-M149 |
|  |  |
|  |  |
| Galactomannan-1 | [CCRC-M75](http://glycomics.ccrc.uga.edu/wall2/jsp/abdetails.jsp?abnumber=133&abname=CCRC-M75) |
|  | [CCRC-M70](http://glycomics.ccrc.uga.edu/wall2/jsp/abdetails.jsp?abnumber=61&abname=CCRC-M70) |
|  | [CCRC-M74](http://glycomics.ccrc.uga.edu/wall2/jsp/abdetails.jsp?abnumber=134&abname=CCRC-M74) |
|  |  |
| Galactomannan-2 | CCRC-M166 |
|  | CCRC-M168 |
|  | CCRC-M174 |
|  | CCRC-M175 |
|  |  |
|  |  |
| Glucomannan | CCRC-M169 |
|  | CCRC-M170 |
|  |  |
|  |  |
| β-Glucan | [LAMP](http://glycomics.ccrc.uga.edu/wall2/jsp/abdetails.jsp?abnumber=47&abname=LAMP2H12H7) |
|  | [BG1](http://glycomics.ccrc.uga.edu/wall2/jsp/abdetails.jsp?abnumber=48&abname=BG1) |
|  |  |
|  |  |
| HG Backbone-1 | [CCRC-M131](http://glycomics.ccrc.uga.edu/wall2/jsp/abdetails.jsp?abnumber=181&abname=CCRC-M131) |
|  | [CCRC-M38](http://glycomics.ccrc.uga.edu/wall2/jsp/abdetails.jsp?abnumber=45&abname=CCRC-M38) |
|  | [JIM5](http://glycomics.ccrc.uga.edu/wall2/jsp/abdetails.jsp?abnumber=14&abname=JIM5) |
|  |  |
|  |  |
| HG Backbone-2 | [JIM136](http://glycomics.ccrc.uga.edu/wall2/jsp/abdetails.jsp?abnumber=57&abname=JIM136) |
|  | [JIM7](http://glycomics.ccrc.uga.edu/wall2/jsp/abdetails.jsp?abnumber=13&abname=JIM7) |
|  |  |
|  |  |
| RG-I Backbone | [CCRC-M69](http://glycomics.ccrc.uga.edu/wall2/jsp/abdetails.jsp?abnumber=172&abname=CCRC-M69) |
|  | [CCRC-M35](http://glycomics.ccrc.uga.edu/wall2/jsp/abdetails.jsp?abnumber=66&abname=CCRC-M35) |
|  | [CCRC-M36](http://glycomics.ccrc.uga.edu/wall2/jsp/abdetails.jsp?abnumber=37&abname=CCRC-M36) |
|  | [CCRC-M14](http://glycomics.ccrc.uga.edu/wall2/jsp/abdetails.jsp?abnumber=67&abname=CCRC-M14) |
|  | [CCRC-M129](http://glycomics.ccrc.uga.edu/wall2/jsp/abdetails.jsp?abnumber=104&abname=CCRC-M129) |
|  | [CCRC-M72](http://glycomics.ccrc.uga.edu/wall2/jsp/abdetails.jsp?abnumber=135&abname=CCRC-M72) |
|  |  |
|  |  |
| Linseed Mucilage RG-I | [JIM3](http://glycomics.ccrc.uga.edu/wall2/jsp/abdetails.jsp?abnumber=79&abname=JIM1) |
|  | [CCRC-M40](http://glycomics.ccrc.uga.edu/wall2/jsp/abdetails.jsp?abnumber=83&abname=CCRC-M40) |
|  | CCRC-M161 |
|  | CCRC-M164 |
|  |  |
|  |  |
| Physcomitrella Pectin | [CCRC-M98](http://glycomics.ccrc.uga.edu/wall2/jsp/abdetails.jsp?abnumber=115&abname=CCRC-M98) |
|  | [CCRC-M94](http://glycomics.ccrc.uga.edu/wall2/jsp/abdetails.jsp?abnumber=118&abname=CCRC-M94) |
|  |  |
|  |  |
| RG-Ia | [CCRC-M5](http://glycomics.ccrc.uga.edu/wall2/jsp/abdetails.jsp?abnumber=81&abname=CCRC-M5) |
|  | [CCRC-M2](http://glycomics.ccrc.uga.edu/wall2/jsp/abdetails.jsp?abnumber=8&abname=CCRC-M2) |
|  |  |
|  |  |
| RG-Ib | [JIM137](http://glycomics.ccrc.uga.edu/wall2/jsp/abdetails.jsp?abnumber=58&abname=JIM137) |
|  | [JIM101](http://glycomics.ccrc.uga.edu/wall2/jsp/abdetails.jsp?abnumber=55&abname=JIM101) |
|  | [CCRC-M61](http://glycomics.ccrc.uga.edu/wall2/jsp/abdetails.jsp?abnumber=138&abname=CCRC-M61) |
|  | [CCRC-M30](http://glycomics.ccrc.uga.edu/wall2/jsp/abdetails.jsp?abnumber=33&abname=CCRC-M30) |
|  |  |
|  |  |
| RG-Ic | [CCRC-M23](http://glycomics.ccrc.uga.edu/wall2/jsp/abdetails.jsp?abnumber=92&abname=CCRC-M23) |
|  | [CCRC-M17](http://glycomics.ccrc.uga.edu/wall2/jsp/abdetails.jsp?abnumber=74&abname=CCRC-M17) |
|  | [CCRC-M19](http://glycomics.ccrc.uga.edu/wall2/jsp/abdetails.jsp?abnumber=0&abname=CCRC-M19) |
|  | [CCRC-M18](http://glycomics.ccrc.uga.edu/wall2/jsp/abdetails.jsp?abnumber=0&abname=CCRC-M18) |
|  | [CCRC-M56](http://glycomics.ccrc.uga.edu/wall2/jsp/abdetails.jsp?abnumber=141&abname=CCRC-M56) |
|  | [CCRC-M16](http://glycomics.ccrc.uga.edu/wall2/jsp/abdetails.jsp?abnumber=73&abname=CCRC-M16) |
|  |  |
|  |  |
| RG-I/Arabinogalactan | [CCRC-M60](http://glycomics.ccrc.uga.edu/wall2/jsp/abdetails.jsp?abnumber=139&abname=CCRC-M60) |
|  | [CCRC-M41](http://glycomics.ccrc.uga.edu/wall2/jsp/abdetails.jsp?abnumber=82&abname=CCRC-M41) |
|  | [CCRC-M80](http://glycomics.ccrc.uga.edu/wall2/jsp/abdetails.jsp?abnumber=128&abname=CCRC-M80) |
|  | [CCRC-M79](http://glycomics.ccrc.uga.edu/wall2/jsp/abdetails.jsp?abnumber=129&abname=CCRC-M79) |
|  | [CCRC-M44](http://glycomics.ccrc.uga.edu/wall2/jsp/abdetails.jsp?abnumber=68&abname=CCRC-M44) |
|  | [CCRC-M33](http://glycomics.ccrc.uga.edu/wall2/jsp/abdetails.jsp?abnumber=75&abname=CCRC-M33) |
|  | [CCRC-M32](http://glycomics.ccrc.uga.edu/wall2/jsp/abdetails.jsp?abnumber=35&abname=CCRC-M32) |
|  | [CCRC-M13](http://glycomics.ccrc.uga.edu/wall2/jsp/abdetails.jsp?abnumber=43&abname=CCRC-M13) |
|  | [CCRC-M42](http://glycomics.ccrc.uga.edu/wall2/jsp/abdetails.jsp?abnumber=86&abname=CCRC-M42) |
|  | [CCRC-M24](http://glycomics.ccrc.uga.edu/wall2/jsp/abdetails.jsp?abnumber=93&abname=CCRC-M24) |
|  | [CCRC-M12](http://glycomics.ccrc.uga.edu/wall2/jsp/abdetails.jsp?abnumber=71&abname=CCRC-M12) |
|  | [CCRC-M7](http://glycomics.ccrc.uga.edu/wall2/jsp/abdetails.jsp?abnumber=3&abname=CCRC-M7) |
|  | [CCRC-M77](http://glycomics.ccrc.uga.edu/wall2/jsp/abdetails.jsp?abnumber=131&abname=CCRC-M77) |
|  | [CCRC-M25](http://glycomics.ccrc.uga.edu/wall2/jsp/abdetails.jsp?abnumber=84&abname=CCRC-M25) |
|  | [CCRC-M9](http://glycomics.ccrc.uga.edu/wall2/jsp/abdetails.jsp?abnumber=69&abname=CCRC-M9) |
|  | [CCRC-M128](http://glycomics.ccrc.uga.edu/wall2/jsp/abdetails.jsp?abnumber=183&abname=CCRC-M128) |
|  | [CCRC-M126](http://glycomics.ccrc.uga.edu/wall2/jsp/abdetails.jsp?abnumber=184&abname=CCRC-M126) |
|  | [CCRC-M134](http://glycomics.ccrc.uga.edu/wall2/jsp/abdetails.jsp?abnumber=102&abname=CCRC-M134) |
|  | [CCRC-M125](http://glycomics.ccrc.uga.edu/wall2/jsp/abdetails.jsp?abnumber=185&abname=CCRC-M125) |
|  | [CCRC-M123](http://glycomics.ccrc.uga.edu/wall2/jsp/abdetails.jsp?abnumber=187&abname=CCRC-M123) |
|  | [CCRC-M122](http://glycomics.ccrc.uga.edu/wall2/jsp/abdetails.jsp?abnumber=188&abname=CCRC-M122) |
|  | [CCRC-M121](http://glycomics.ccrc.uga.edu/wall2/jsp/abdetails.jsp?abnumber=189&abname=CCRC-M121) |
|  | [CCRC-M112](http://glycomics.ccrc.uga.edu/wall2/jsp/abdetails.jsp?abnumber=169&abname=CCRC-M112) |
|  | [CCRC-M21](http://glycomics.ccrc.uga.edu/wall2/jsp/abdetails.jsp?abnumber=88&abname=CCRC-M21) |
|  | [JIM131](http://glycomics.ccrc.uga.edu/wall2/jsp/abdetails.jsp?abnumber=94&abname=JIM131) |
|  | [CCRC-M22](http://glycomics.ccrc.uga.edu/wall2/jsp/abdetails.jsp?abnumber=46&abname=CCRC-M22) |
|  | [JIM132](http://glycomics.ccrc.uga.edu/wall2/jsp/abdetails.jsp?abnumber=56&abname=JIM132) |
|  | [JIM1](http://glycomics.ccrc.uga.edu/wall2/jsp/abdetails.jsp?abnumber=79&abname=JIM1) |
|  | [CCRC-M15](http://glycomics.ccrc.uga.edu/wall2/jsp/abdetails.jsp?abnumber=72&abname=CCRC-M15) |
|  | [CCRC-M8](http://glycomics.ccrc.uga.edu/wall2/jsp/abdetails.jsp?abnumber=29&abname=CCRC-M8) |
|  | [JIM16](http://glycomics.ccrc.uga.edu/wall2/jsp/abdetails.jsp?abnumber=62&abname=JIM16) |
|  |  |
|  |  |
| Arabinogalactan-1 | [JIM93](http://glycomics.ccrc.uga.edu/wall2/jsp/abdetails.jsp?abnumber=117&abname=JIM93) |
|  | [JIM94](http://glycomics.ccrc.uga.edu/wall2/jsp/abdetails.jsp?abnumber=95&abname=JIM94) |
|  | [JIM11](http://glycomics.ccrc.uga.edu/wall2/jsp/abdetails.jsp?abnumber=41&abname=JIM11) |
|  | [MAC204](http://glycomics.ccrc.uga.edu/wall2/jsp/abdetails.jsp?abnumber=23&abname=MAC204) |
|  | [JIM20](http://glycomics.ccrc.uga.edu/wall2/jsp/abdetails.jsp?abnumber=91&abname=JIM20) |
|  |  |
|  |  |
| Arabinogalactan-2 | [JIM14](http://glycomics.ccrc.uga.edu/wall2/jsp/abdetails.jsp?abnumber=31&abname=JIM14) |
|  | [JIM19](http://glycomics.ccrc.uga.edu/wall2/jsp/abdetails.jsp?abnumber=44&abname=JIM19) |
|  | [JIM12](http://glycomics.ccrc.uga.edu/wall2/jsp/abdetails.jsp?abnumber=191&abname=JIM12) |
|  | [CCRC-M133](http://glycomics.ccrc.uga.edu/wall2/jsp/abdetails.jsp?abname=CCRC-M133) |
|  | [CCRC-M107](http://glycomics.ccrc.uga.edu/wall2/jsp/abdetails.jsp?abnumber=166&abname=CCRC-M107) |
|  |  |
|  |  |
| Arabinogalactan-3 | [JIM4](http://glycomics.ccrc.uga.edu/wall2/jsp/abdetails.jsp?abnumber=40&abname=JIM4) |
|  | [CCRC-M31](http://glycomics.ccrc.uga.edu/wall2/jsp/abdetails.jsp?abnumber=34&abname=CCRC-M31) |
|  | [JIM17](http://glycomics.ccrc.uga.edu/wall2/jsp/abdetails.jsp?abnumber=39&abname=JIM17) |
|  | [CCRC-M26](http://glycomics.ccrc.uga.edu/wall2/jsp/abdetails.jsp?abnumber=85&abname=CCRC-M26) |
|  | [JIM15](http://glycomics.ccrc.uga.edu/wall2/jsp/abdetails.jsp?abnumber=32&abname=JIM15) |
|  | [JIM8](http://glycomics.ccrc.uga.edu/wall2/jsp/abdetails.jsp?abnumber=80&abname=JIM8) |
|  | [CCRC-M85](http://glycomics.ccrc.uga.edu/wall2/jsp/abdetails.jsp?abnumber=121&abname=CCRC-M85) |
|  | [CCRC-M81](http://glycomics.ccrc.uga.edu/wall2/jsp/abdetails.jsp?abnumber=127&abname=CCRC-M81) |
|  | [MAC266](http://glycomics.ccrc.uga.edu/wall2/jsp/abdetails.jsp?abnumber=98&abname=MAC266) |
|  | [PN 16.4B4](http://glycomics.ccrc.uga.edu/wall2/jsp/abdetails.jsp?abnumber=11&abname=PN%2016.4B4) |
|  |  |
|  |  |
| Arabinogalactan-4 | [MAC207](http://glycomics.ccrc.uga.edu/wall2/jsp/abdetails.jsp?abnumber=22&abname=MAC207) |
|  | [JIM133](http://glycomics.ccrc.uga.edu/wall2/jsp/abdetails.jsp?abnumber=96&abname=JIM133) |
|  | [JIM13](http://glycomics.ccrc.uga.edu/wall2/jsp/abdetails.jsp?abnumber=30&abname=JIM13) |
|  | [CCRC-M92](http://glycomics.ccrc.uga.edu/wall2/jsp/abdetails.jsp?abnumber=119&abname=CCRC-M92) |
|  | [CCRC-M91](http://glycomics.ccrc.uga.edu/wall2/jsp/abdetails.jsp?abnumber=120&abname=CCRC-M91) |
|  | [CCRC-M78](http://glycomics.ccrc.uga.edu/wall2/jsp/abdetails.jsp?abnumber=130&abname=CCRC-M78) |
|  |  |
|  |  |
| Unidentified | [MAC265](http://glycomics.ccrc.uga.edu/wall2/jsp/abdetails.jsp?abnumber=97&abname=MAC265) |
|  | [CCRC-M97](http://glycomics.ccrc.uga.edu/wall2/jsp/abdetails.jsp?abnumber=116&abname=CCRC-M97) |
|  |  |

**References**

1. Pattathil S, Avci U, Baldwin D, Swennes AG, McGill JA, Popper Z, Bootten T, Albert A, Davis RH, Chennareddy C: **A comprehensive toolkit of plant cell wall glycan-directed monoclonal antibodies**. *Plant physiology* 2010, **153**(2):514-525.

2. Pattathil S, Avci U, Miller JS, Hahn MG: **Immunological approaches to plant cell wall and biomass characterization: glycome profiling**. In: *Biomass Conversion.* Springer; 2012: 61-72.
